# Quantitative Visions of Reality at the Tick-Host Interface: Biochemistry, Genomics, Proteomics, and Transcriptomics as Measures of Complete Inventories of the Tick Sialoverse

**DOI:** 10.3389/fcimb.2020.574405

**Published:** 2020-09-11

**Authors:** Ben J. Mans

**Affiliations:** ^1^Epidemiology, Parasites and Vectors, Agricultural Research Council-Onderstepoort Veterinary Research, Pretoria, South Africa; ^2^Department of Veterinary Tropical Diseases, University of Pretoria, Pretoria, South Africa; ^3^Department of Life and Consumer Sciences, University of South Africa, Pretoria, South Africa

**Keywords:** tick, sialome, sialoverse, proteome, transcriptome, salivary gland

## Abstract

Species have definitive genomes. Even so, the transcriptional and translational products of the genome are dynamic and subject to change over time. This is especially true for the proteins secreted by ticks at the tick-host feeding interface that represent a complex system known as the sialoverse. The sialoverse represent all of the proteins derived from tick salivary glands for all tick species that may be involved in tick-host interaction and the modulation of the host's defense mechanisms. The current study contemplates the advances made over time to understand and describe the complexity present in the sialoverse. Technological advances at given periods in time allowed detection of functions, genes, and proteins enabling a deeper insight into the complexity of the sialoverse and a concomitant expansion in complexity with as yet, no end in sight. The importance of systematic classification of the sialoverse is highlighted with the realization that our coverage of transcriptome and proteome space remains incomplete, but that complete descriptions may be possible in the future. Even so, analysis and integration of the sialoverse into a comprehensive understanding of tick-host interactions may require further technological advances given the high level of expected complexity that remains to be uncovered.

## Introduction

**Figure 1:**
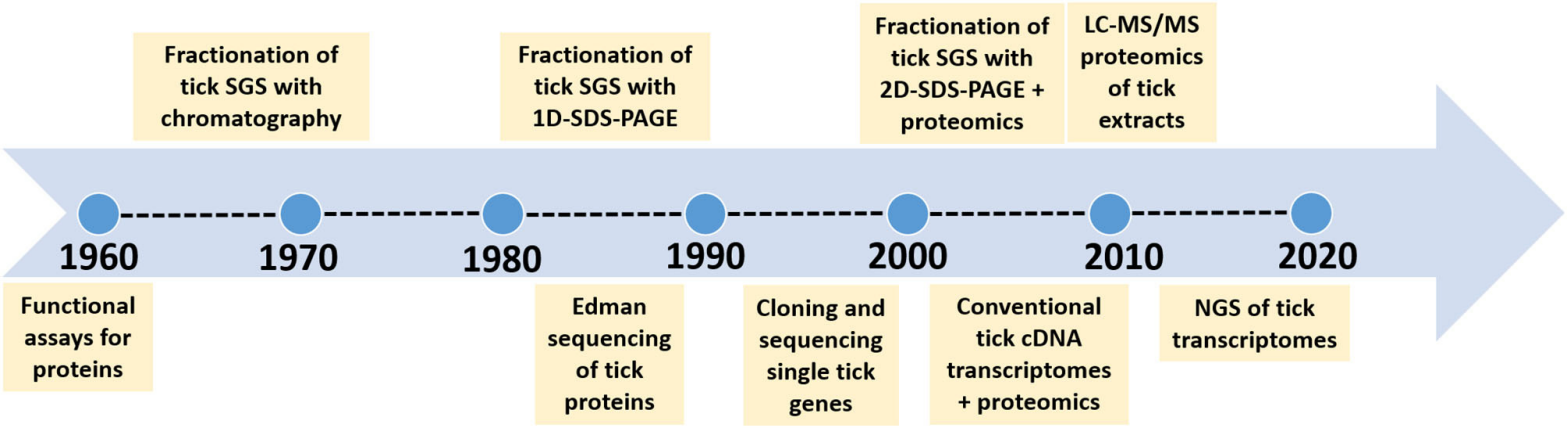
Time line to indicate the major technological advances that improved our estimation of complexity of salivary gland proteins.

Organisms are finite creatures in space and time with genomes that are definitive for a given species. As such, within the population of organisms that comprise a specific species, their genomes will be similar in identity and synteny over ~95–99% of the total genome. This last 3–5% of intra-species diversity comprise alleles, epigenetic differences, single nucleotide polymorphisms and lost or rearranged genes that forms the basis for phenotypic or strain differences. The contribution of the microbiome that forms with the tick genome the hologenome, adds considerable diversity at individual and population level (Díaz-Sánchez et al., 2019). Horizontal transfer may occur from the microbiome to the core genome, but in this case these transferred genes would then become part of the core genome. However, the hologenome is not considered in the current study, since these are exogenous variable factors that may not be part of the core genetic component of a species. The core genetic component comprises not only the genome, but its translational aspects such as the transcriptome and proteome, which are more dynamic in nature, since the final products such as mRNA, proteins and other metabolites show tissue-specific and temporal expression patterns, different half-life's and concentration levels. The impact of concentration levels of proteins and metabolites at the tick feeding site has recently been explored and shown to be critical for effective functioning (Mans, 2019). An extensive summary of functions for tick proteins involved at the tick-host interface was also presented previously (Mans, 2016, 2019; Mans et al., 2016). The acquisition of new functions by gene duplication has also been explored and shown to play an important part in the expansion of gene families in ticks (Mans et al., 2017). The current review considers the progress on quantitative description of molecules at the feeding site and addresses the question on how our understanding of the number of proteins and functions at the feeding site has changed over time due to technological advances and experimental design (Figure 1).

## Complexity Measured by Morphology and Histochemistry

The recognition that salivary glands have a complex morphology composed of non-secretory and secretory acini (Robinson and Davidson, 1913), as well as multiple cell types within secretory acini, have been well-recognized once cell staining and tissue sectioning could be applied to tick salivary glands (Till, 1959; Chinery, 1965; Balashov, 1972). While soft ticks have a single granular acini, female hard ticks have two types (II and III), while male hard ticks also have a fourth type (IV) (Till, 1959; Roshdy, 1972). Granular acini from both soft and hard ticks all have multiple cell types, with both histochemical staining and immunolocalization indicating that different cells show differential expression of proteins (Roshdy, 1972; Coons and Roshdy, 1973; Mans et al., 2004). Morphological observation was therefore the first indication that salivary glands have higher complexity than may be expected from a simple secretory organ that may be likened to a bag of proteins.

## Complexity Measured by Functional Activity

In the absence of fractionation, estimates of complexity may be obtained by measuring unique functions in salivary glands, salivary gland secretions (SGS) or salivary gland extracts (SGE) using empirical methods. As such, it was indicated that tick salivary glands possess numerous functionalities suggesting a complex organ with many functions active at the tick feeding site (Neitz and Vermeulen, 1987). These included enzymatic functions, enzyme inhibitory functions, anticoagulants, cement, toxins and immunogens. Many proteins responsible for these functions were later isolated and identified from numerous tick species, with ~120 experimentally verified tick protein functions to date (reviewed in Mans, 2019). The difficulty in complexity estimation using functional characterization is the sheer immensity of potential known and unknown functions and the amount of effort necessary to characterize these functions (Ribeiro and Arcà, 2009; Mans, 2016).

## Complexity Measured by Counting Peaks

Kaire (1966) performed the first chromatographic separation of tick proteins from whole tick extracts to purify the neurotoxin from the Australian paralysis tick, *Ixodes holocyclus* (Neumann, 1899). However, the first fractionation of tick salivary proteins arguably occurred in the 1960 and 1970s when Neitz purified the first toxins from SGS from the soft tick *Ornithodoros savignyi* (Audouin, 1826) (sand tampan), now *Ornithodoros kalahariensis* (Neitz et al., 1969; Howell et al., 1975; Bakkes et al., 2018). The fractionation used combinations of size exclusion, ion exchange and iso-electric focusing methods available at the time. Estimation of the number of proteins that was present in the SGS is dependent on counting protein peaks with corresponding assumptions regarding the complexity of protein numbers within such peaks. Looking at the work of Neitz et al. (1969) in retrospect and doing back of the envelope estimations suggest a low complexity mixture of <100 proteins. For this specific tick species, even with modern HPLC methods, 1-dimensional sodium dodecyl sulfate poly-acrylamide electrophoresis (1D-SDS-PAGE), or 2-dimensional SDS-PAGE (2D-SDS-PAGE), and using SGE rather than SGS (SGE contain both secretory and non-secretory proteins, while SGS should be more abundant in secretory proteins), the complexity estimates did not change much (Gaspar et al., 1995, 1996; Mans et al., 2001, 2002a; Mans and Neitz, 2004a). For other soft tick species the generalization that <200 proteins may be observed in SGE using HPLC, 1D-SDS-PAGE or 2D-SDS-PAGE also holds (Oleaga et al., 2007; Francischetti et al., 2008a,b; Mans et al., 2008).

For the hard tick *Amblyomma hebraeum* (Koch, 1844) (South African Bont tick), chromatography and microzone electrophoresis suggested a low complexity mixture (Neitz and Vermeulen, 1987). However, it should be noted that the latter study again used SGS from a single feeding stage (partially fed). Whether SGS obtained from chemical induction represents salivary gland complexity has been addressed previously (Mans, 2019), and reservations exist whether such a secretion can be used as accurate measurement of salivary gland protein complexity, since different stimulants results in different proteins secreted (Oliveira et al., 2013).

## Complexity Measurement by Chromatographic Fractionation, Edman and Gene Sequencing

Chromatographic fractionation of whole body, SGS or SGE enabled purification of proteins for functional characterization, while development of automated Edman degradation allowed for the first N-terminal amino acid sequences generated for tick salivary gland proteins (Neitz et al., 1983). This approach allowed for the full-length Edman sequencing of a variety of small anticoagulants and platelet aggregation inhibitors (Waxman et al., 1990; Karczewski et al., 1994; Wang et al., 1996). Generation of N-terminal or internal Edman sequences also allowed for the design of degenerate primers or probes that enabled cloning of gene sequences (Keller et al., 1993; Waxman and Connolly, 1993; Gaspar et al., 1996; Joubert et al., 1998; Nienaber et al., 1999; Paesen et al., 1999; Bergman et al., 2000; Valenzuela et al., 2000; Mans et al., 2001, 2002b,c, 2003; Narasimhan et al., 2002; Mulenga et al., 2003). This approach led to generation of ~30 tick salivary gland sequences by 2003. Our insights into salivary gland complexity were heavily skewed at this point in time by the limited number of functions known (Mans and Neitz, 2004b). Even so, we still remain with very few empirically confirmed functions to date (Mans, 2019), although function assignment by homology allowed thousands of potential functions to be assigned to tick proteins (Mans et al., 2016; Pienaar et al., 2018; Ribeiro and Mans, 2020).

## Complexity Measured by Counting Bands

The development of high resolution 1D-SDS-PAGE by Laemmli (1970) ushered in a new era of estimating complexity, allowing in theory to differentiate proteins with molecular mass differences of <1,000 Da. Linked with high resolution gel photography, this led to the first glimpses of salivary complexity in the Lone Star tick *Amblyomma americanum* (Linnaeus, 1758) (McSwain et al., 1982). This study was significant, since it analyzed changes in protein patterns over the course of feeding and showed that differential expression does indeed occur. Counting of the bands indicated at least 100 proteins with different molecular masses. Other studies soon followed with similar estimates in complexity for *A. hebraeum*, the brown ear tick *Rhipicephalus appendiculatus* (Neumann, 1901) and the red-legged tick *Rhipicephalus evertsi evertsi* (Neumann, 1897; Neitz and Gothe, 1986; Viljoen et al., 1986; Wang and Nuttall, 1994; Wang et al., 1999, 2001). At this point in time 1D-SDS-PAGE promised a glimpse at salivary gland protein complexity. However, our ability to identify these proteins with proteomics was not yet at a technological advanced level that would enable this (Rabilloud, 2020). The limitation of 1D-SDS-PAGE to reveal complexity was also limited by the resolution afforded and the fact that members of the same protein family would have very similar molecular weights, even if they have different iso-electric points resulting in underestimation of complexity. As such, 100 bands may very well indicate a 100 protein families rather than a 100 proteins.

## Complexity Measured by Counting Spots and Identification by Proteomic Analysis

The advent of 2D-SDS-PAGE that included iso-electric focusing in the first dimension followed by 1D-SDS-PAGE in the second dimension (O'Farrell, 1975), again revolutionized the concept of fractionation of complex mixtures. This produced protein expression maps that correlated with protein iso-electric point and molecular weight represented by spots on a gel. In ticks, the first 2D-SDS-PAGE images were published in 2001 for the deer tick *Ixodes scapularis* (Say, 1821) and *O. kalahariensis* (Das et al., 2001; Mans et al., 2001). Analysis of female tick salivary gland extract from *O. kalahariensis* indicated ~100 proteins on a 2D-electropherogram. The major proteins present in this electropherogram could be confirmed to be soft tick proteins based on protein purification, peptide mass fingerprinting and tick sequences obtained via painstaking single gene sequencing (Mans et al., 2001; Mans and Neitz, 2004a). The 2D-electropherogram for *I. scapularis* revealed almost 500 spots in SGE (Das et al., 2001), and this complexity was again confirmed (Narasimhan et al., 2007). This latter study also indicated that the proteome changes over the time of feeding using Differential 2D Fluorescence Gel Electrophoresis (DIGE).

The first 2D-SDS-PAGE followed by proteomic analysis was performed for the SGS of *A. americanum* and the Gulf Coast tick, *Amblyomma maculatum* (Koch, 1844) (Madden et al., 2002). This revealed ~100–200 protein spots on the 2D-electropherogram. Surprisingly, the majority of spots reacted with anti-sheep serum suggesting that they were host derived. Of 16 prominent spots picked for peptide mass fingerprinting, one spot was identified as a tick protein and five spots as host proteins. It should be considered that at this point only 20 protein sequences were available for *A. americanum* and only 287 tick proteins were present in Genbank, which may explain the low number of tick proteins identified. This highlighted the impact that the lack of sequence coverage may have on proteomic detection. Other factors that limited the use of 2D-SDS-PAGE as method for saliva analysis is the high salt concentration found in tick saliva that necessitate extra desalting steps (Madden et al., 2002).

## Complexity Measured by CDNA Library Sequencing and Proteomics

The advent of complementary DNA (cDNA) library synthesis (Chenchik et al., 1996; Lukyanov et al., 1997) and propagation as unique clones using phage packaging (Kretz et al., 1989), allowed direct sequencing of mRNA derived genes that gave new insight into salivary gland and transcriptome complexity (Table 1). The first cDNA libraries constructed were expression libraries used to identify antigenic proteins recognized by host antiserum and subsequent sequencing of immunogenic clones (Das et al., 2000, 2001; Bishop et al., 2002). This yielded 14 immunogenic sequences for *I. scapularis* (Das et al., 2001). Larger scale, studies identified 895 immuno-proteins using similar approached in *A. americanum* (Radulović et al., 2014). Alternative approaches included the use of probes to screen a cDNA library for homologous genes (Sangamnatdej et al., 2002).

**Table 1:** Statistics for conventional cDNA library sequencing.

**Species**	**Clones**	**Clusters**	**Genbank**	**Secretory**	**Proteomics**	**Proteomic coverage (%)**	**References**
*Ixodes ricinus*	96	27	29	–	–	–	Leboulle et al., 2002
*Ixodes scapularis*	735	410	87	102	19 tick; 2 host	21.8	Valenzuela et al., 2002a
*Amblyomma variegatum*	3,992	2,109	–	–	–	–	Nene et al., 2002
*Rhipicephalus appendiculatus*	19,006	7,359	19,046	–	–	–	Nene et al., 2004
*Rhipicephalus microplus*	11,590	8,270	–	–	–	–	Guerrero et al., 2005
*Ixodes pacificus*	1,068	557	120	83	–	–	Francischetti et al., 2005
*Ixodes scapularis*	8,150	3,020	514	863	–	–	Ribeiro et al., 2006
*Dermacentor andersoni*	1,440	762	1,270	239	–	–	Alarcon-Chaidez et al., 2007
*Ornithodoros parkeri*	1,529	649	158	130	37	23.4	Francischetti et al., 2008a
*Ornithodoros coriaceus*	1,089	726	105	127	39	37	Francischetti et al., 2008b
*Argas monolakensis*	3,087	1,472	193	127	35	18.1	Mans et al., 2008
*Ixodes ricinus*	1,881	1,274	511	129	–	–	Chmelar et al., 2008
*Amblyomma americanum*	6,160	4,577	141	193	–	–	Aljamali et al., 2009
*Rhipicephalus sanguineus*	2,034	1,024	217	219	21 tick; 56 host	9.7	Anatriello et al., 2010; Oliveira et al., 2013
*Amblyomma variegatum*	3,992	2,077	605	379	170	28.0	Ribeiro et al., 2011
*Hyalomma rufipes*	2,084	1,167	98	255	72 tick, 22 host	73.4	Francischetti et al., 2011
*Antricola delacruzi*	1,147	923	38	115	–	–	Ribeiro et al., 2012
*Amblyomma americanum*	15,390	12,319	14,958	–	–	–	Gibson et al., 2013

*Indicated are the tick species, the number of clones sequenced by Sanger sequencing, the non-redundant clusters obtained after clusterization of sequenced clones, final number of genes submitted to Genbank, the number of secretory proteins detected, the number of tick or host proteins detected using proteomics and the proteomic coverage in percentage of proteins detected relative to the genes submitted to Genbank. Transcriptomes are organized based on publication date*.

The first tick salivary gland cDNA libraries synthesized and systematically sequenced using Sanger sequencing yielded 36 sequences for the castor bean tick *Ixodes ricinus* (Linnaeus, 1758) and 87 sequences for *I. scapularis*, respectively (Leboulle et al., 2002; Valenzuela et al., 2002a). The latter study also identified 19 tick proteins and 2 host proteins using Western blot analysis followed by Edman sequencing and used these sequences to identify corresponding cDNA sequences (Valenzuela et al., 2002a). This already highlighted the importance of having species specific transcriptome sequences available. It also indicated the utility of cDNA library sequencing, when the same cDNA data was used to predict function by homology and subsequent functional characterization of recombinant proteins, leading to the discovery of the anti-coagulant Ixolaris (Francischetti et al., 2002) and a fibrinogenase metalloprotease (Francischetti et al., 2003). The study by Valenzuela et al. (2002a) was further significant in that it represented the first systematic high-throughput randomized sequencing of tick salivary gland cDNA clones (735 clones sequenced that clustered in 410 clusters and yielded 87 unique full-length sequences), that also attempted a systematic classification of salivary transcripts into groups or protein families (7 groups plus various singletons). Secretory proteins accounted for 102 clusters and 310 of the clones sequenced. It also introduced for the first time the term sialome (from the Greek σíελoç = saliva) to describe the set of mRNA and proteins expressed in the salivary glands of ticks. A novel algorithm of deconvoluting Edman sequences obtained from crude SGE fractionated on 1D-SDS-PAGE was also presented, that allowed mixed Edman sequences to be matched to the transcriptome (Valenzuela et al., 2002b). This technique was subsequently successfully used in various transcriptome studies to identify abundant proteins (Francischetti et al., 2008a,b, 2011; Mans et al., 2008; Ribeiro et al., 2011).

Shortly after this groundbreaking study, more studies started to report on systematic conventional cDNA library transcriptome Sanger sequencing. For the Tropical Bont tick, *Amblyomma variegatum* (Fabricius, 1794), the salivary gland transcriptome was presented as the AvGI (*A. variegatum* gene index) and sequenced 3,992 clones of which 2,109 was non-redundant and 822 showed similarity to sequences in the database (Nene et al., 2002). No proteomics was performed, and no systematic classification presented, although a high-level gene ontology was assigned. A number of years later the same dataset was re-analyzed with the aim at classifying the genes and resulted in 3,985 EST sequences clustering into 2,077 contigs, of which 605 was submitted to Genbank (Ribeiro et al., 2011). The secretory proteins (379) were classified into 21 groups or families and a 1D-SDS-PAGE fractionation of SGE, from which 24 bands were analyzed by proteomics resulted in the identification of 170 proteins that represented 28% of the EST database submitted to Genbank.

For *R. appendiculatus*, 9,162 clones from an uninfected cDNA library and 9,844 clones from a *Theileria parva* infected cDNA library were sequenced (Nene et al., 2004). This resulted in 7,359 non-redundant sequences. Secretory genes were not classified in any systematic manner although gene ontology was presented. No proteomics was performed. This was followed by the *Rhipicephalus microplus* (Canestrini, 1888) gene index (BmGI) (Asian Blue tick) that was a whole body transcriptome where 11,590 clones were sequenced from a normalized cDNA library resulting in 8,270 unique sequences (Guerrero et al., 2005). A proteomic analysis of larval extract fractionated using 2D-SDS-PAGE, from which 20 spots were selected resulted in 18 proteins being identified (Untalan et al., 2005). This indicated the utility of a representative species specific transcriptome databases.

The salivary gland transcriptome of the Western-Blacklegged tick, *Ixodes pacificus* (Cooley and Kohls, 1943) was constructed via sequencing of only 1,068 clones that clustered into 557 contigs (Francischetti et al., 2005). Even though few clones were sequenced, classification of secretory proteins still resulted in 15 groups and 83 secretory proteins, creating the notion that the most abundant proteins and therefore those most important for feeding would still be represented in small scale transcriptome sequencing projects. However, a project that sequenced 8,150 clones from *I. scapularis* from nymphs and adults from various feeding stages resulted in 3,020 contigs and 863 unique secretory proteins (Ribeiro et al., 2006). This study was the first indication that secretory proteins may be present at levels of more than 500 proteins and possibly extending into thousands, suggesting that tick salivary gland diversity may be much more extensive than previously expected. The transcriptome for *I. ricinus* was also described from four different cDNA libraries (unfed, 24 h after attachment, 4 days—partially fed and 7 days—fully engorged) (Chmelar et al., 2008). A total of 1,881 clones were sequenced that clustered into 1,274 clusters of which 511 was submitted to the nucleotide database. Of these 129 was classified as secretory.

Alarcon-Chaidez et al. (2007) described the salivary gland transcriptome of the Rocky Mountain Wood tick, *Dermacentor andersoni* (Stiles, 1908). The study sequenced 1,440 clones that clustered into 762 unique sequences. Of these ~75% found homologs in the existing databases. The salivary gland transcriptome for *A. americanum* was described by Aljamali et al. (2009). A total of 6,160 clones were sequenced from both non-normalized and normalized libraries and clustered in 4,577 contigs. Of these 141 were submitted as proteins to Genbank. Reanalysis of the nucleotide sequences identified 193 secretory proteins in the dataset (Mans et al., 2016). A follow up study sequenced 15,390 clones that yielded 12,319 unique sequences (Gibson et al., 2013). The study observed that 71% of all sequences generated could not be annotated by function assignment by homology. Possible reasons presented were divergence in the tick lineage, the presence of lineage specific genes and limited genomic resources for ticks that would allow homology assignment. The study did not attempt to assign these genes to secretory families and the high level of non-homologous orphan sequences that did not find any hits to other tick sequences remain surprising.

Anatriello et al. (2010) described the salivary gland transcriptome of the brown dog tick, *Rhipicephalus sanguineus* (Latreille, 1806) and sequenced 2,034 clones that yielded 1,024 non-redundant sequences. Secretory proteins comprised 219 sequences and were classified into 12 protein family classes. A proteomic study was performed of saliva collected by either dopamine or pilocarpine stimulation (Oliveira et al., 2013). Saliva was fractionated on 1D-SDS-PAGE, bands were excised, followed by tryptic digestion and nanoflow reversed-phase liquid chromatography tandem mass spectrometry (nanoRPLC-MS/MS). The transcriptome database previously generated was used for analysis. The protein profiles obtained with dopamine or pilocarpine stimulation differed significantly with pilocarpine stimulation obtaining more bands. For dopamine stimulation only two lipocalins could be identified. For pilocarpine stimulation, 56 rabbit proteins were identified and only 19 tick proteins. Some of the rabbit proteins may have close homologs in ticks, although 16 rabbit proteins were detected that would be mammal specific. Of interest, is that the lipocalins secreted with dopamine were not found in the pilocarpine secretion, which raised again the question regarding representation of secretory proteins obtained with chemical stimulants.

The final ixodid sialome to be analyzed and described in a systematic manner using conventional cDNA sequencing was for the coarse bont-legged tick, *Hyalomma rufipes* (Koch, 1844) (Francischetti et al., 2011). A total of 2,084 clones were sequenced, clustered into 1,167 contigs of which 255 were classified as secretory and 98 proteins were submitted to Genbank. From 20 1D-SDS-PAGE bands analyzed by MS/MS, 72 tick proteins were identified as well as 22 host proteins. The identified proteins comprised 73.4% of the proteins submitted to Genbank.

By 2006 ~12 soft tick salivary gland proteins have been functionally characterized and single genes cloned and sequenced. No salivary gland transcriptomes was systematically described up to this point for soft ticks. A proteomic analysis of SGE from *Ornithodoros erraticus* (Lucas, 1849) and *Ornithodoros moubata* (Murray, 1877), the African hut tampan, based on the proteins then present in the databanks underscored the importance of having species specific transcriptome databases necessary for proteomic analysis (Oleaga et al., 2007). For *O. moubata*, 48 non-redundant proteins were present in the databank and for 40 2D-SDS-PAGE spots analyzed only two proteins (TSGP1 and moubatin) could be identified. For *O. erracticus*, no proteins were available in the databanks and from 54 spots analyzed only 7 proteins were identified. In a follow-up study proteomic analysis of saliva from *O. moubata* was performed using *in situ* tryptic digest followed by liquid chromatography-MS/MS (LC-MS/MS) (Díaz-Martín et al., 2013). At this point in time 75 sequences were available in the databanks for *O. moubata* and proteomic analysis identified 193 proteins. Of these, 99.9% of the protein abundance was accounted for by the major lipocalins: TSGP1, TSGP4, and moubatin, while the remaining 0.1% abundance was accounted for by housekeeping proteins similar to sequences from ticks and insects in the database. The presence of housekeeping proteins may be explained by apocrine secretion or perhaps even salivary gland cell degradation given the low abundance found (Mans et al., 2016). As indicated before, the functional provenance of these low abundance proteins at the tick feeding site needs to be confirmed (Mans, 2019).

In 2008, three argasid salivary transcriptomes were reported for the Mono Lake bird tick *Argas monolakensis* (Schwan et al., 1992), the pajaroello tick *Ornithodoros coriaceus* (Koch, 1844) and the relapsing fever tick *Ornithodoros parkeri* (Cooley, 1936) (Francischetti et al., 2008a,b; Mans et al., 2008). For *A. monolakensis*, 3,087 clones were sequenced and clustered to give 1,472 contigs of which 127 was classified as secretory and 193 proteins were submitted to Genbank (Mans et al., 2008). Analyses of 78 2D-SDS-PAGE spots identified 18 spots and 14 proteins, 14 bands analyzed by Edman sequencing identified 13 proteins, and 116 peaks obtained from HPLC fractionation identified 52 peaks and 27 proteins, resulting in a final number of 35 proteins identified. This comprised 18% of the contigs coding for housekeeping and secretory proteins and 40% of the protein samples analyzed. For *O. coriaceus*, 1,089 clones were sequenced, resulting in 726 contigs, 127 classified as secretory and 105 proteins submitted to Genbank. Edman sequencing of 1D-SDS-PAGE fractions identified 3 proteins, analysis of 60 2D-SDS-PAGE spots identified 7 proteins and proteomic MS/MS analysis of 61 bands from 1D-SDS-PAGE analysis identified 37 proteins, resulting in a final number of 39 proteins (Francischetti et al., 2008b). This comprised 37% of the contigs submitted to Genbank. For *O. parkeri*, 1,529 clones were sequenced, resulting in 649 contigs and 158 proteins submitted to Genbank (Francischetti et al., 2008a). Edman sequencing of 1D-SDS-PAGE bands identified 12 proteins, analysis of 60 2D-SDS-PAGE spots identified 11 proteins and proteomic MS/MS analysis of 51 bands from 1D-SDS-PAGE analysis identified 30 proteins, resulting in a final number of 36 proteins (Francischetti et al., 2008a). This comprised 22.7% of the contigs and 42% of the proteomic fractions analyzed. All of the proteins were secretory and the majority belonged to the 5′-nucleotidase (apyrase), basic pancreatic trypsin inhibitor (BPTI), basic tails secretory (BTSP), cystatin, and lipocalin families.

The results obtained for the argasid sialomes indicated that conventional cDNA libraries sequenced at low levels may not represent all proteins present, but that the proteome also do not represent all transcripts found in the sialome. It also indicated that different methodologies may identify different protein sets and are therefore complementary. Even so, it also made the case that specific transcriptomes contribute toward a higher rate of protein identification. It also indicated that for all of these sialomes the major abundant proteins identified were classified as secretory, notably belonging to the BPTI, BTSP and lipocalin families. It also suggested that soft tick sialomes may represent lower complexity than ixodids. Reasons for this may be traced to the short feeding events of soft ticks, where feeding occurs within a few minutes to hours rather than days (Mans and Neitz, 2004b). This result in secretion of a bolus of salivary material and not the differential expression patterns observed for ixodid ticks (McSwain et al., 1982; Wang and Nuttall, 1994).

The salivary transcriptome for female *Antricola delacruzi* (Estrada-Peña et al., 2004) was also described using conventional cDNA library sequencing (Ribeiro et al., 2012). A total of 1,147 clones were sequenced, resulting in 923 contigs, 115 annotated as secretory proteins with 38 proteins submitted to Genbank. The transcriptome differed completely from those of other soft tick species where the major soft tick protein families are BPTI, BTSP and lipocalin families. Instead, ferritin, mucins with chitin-binding domains and TIL-domain-containing proteins were abundant. A reason for this may lie in the fact that *Antricola* adults do not feed on blood. The larvae and possibly nymphs do feed on blood (Estrada-Peña et al., 2008), and it may be expected that these life stages may present the more canonical protein families found in blood-feeding argasids.

Conventional Sanger sequencing of cDNA libraries to generate insights into salivary gland transcriptome composition contributed tremendously to our knowledge of salivary gland complexity, even if in the final analysis it became clear that this approach was not exhaustively descriptive or quantitative. This period saw the classification of secretory salivary gland proteins into well-described families (Francischetti et al., 2009), the detection and description of abundant secretory proteins and the observation that protein families are conserved among tick families. It showed that ticks possess their own lineage specific protein repertoires, that gene duplication plays a significant role in creating lineage specific expansions and that the salivary gland protein repertoire may be much more complex than we expected originally (Mans et al., 2008). The data contributed to our ability to detect and identify salivary proteins by proteomic analysis and showed that species-specific sequence databases are crucial for proteomic identification. Where transcriptome data was available, a significant number of genes in the transcriptome could be confirmed by proteomics. This was a golden age in salivary gland discovery and we may have happily continued with this low-level description if the next revolution in sequence technology did not occurred, namely next-generation sequencing.

## Complexity Measured by Next-Generation Sequencing of Transcriptomes and Proteomics

The ability to generate automated high-throughput sequence data that was independent of cDNA library construction was made possible by the development of a wide variety of next-generation sequencing technologies that included Roche 454, Ion Torrent, Illumina HiSeq, and MiSeq technologies (Levy and Myers, 2016). These technologies purified mRNA directly, fragmented and directly sequenced all fragments at the same time resulting in large datasets with millions of reads. The reads are then assembled into contigs using a variety of next-generation algorithms (Martin and Wang, 2011). It enables the complete *de novo* assembly of a whole transcriptomes in the absence of any genome data. It also has the advantage that sequence depth may be converted into sequence coverage, giving an indication of transcript abundance and differential expression.

The first tick salivary gland transcriptome sequenced using these technologies was for *A. maculatum* using the Roche 454 GS FLX titanium pyrosequencing (Karim et al., 2011). The run generated 1,626,969 reads with an average length of 344 bp. An initial 190,646 contigs were assembled, but were reduced to 72,441 using a size cut-off above 149 bp. A final number of 15,814 were analyzed of which 4,849 were submitted to Genbank. An astounding 3,475 secretory proteins were identified with 304 members of the lipocalin family, the latter comprising almost the number of secretory proteins previously found in conventional cDNA libraries. These numbers were orders of magnitude higher than any transcriptome previously produced and hinted a depth of complexity not previously imagined.

The next transcriptome to be described was for *I. ricinus* using a combination of Roche 454 and Illumina technology (Schwarz et al., 2013). For Roche 454, 441,381 reads were generated and for Illumina an astounding 67,703,183 reads for the time. Assembly of the combined reads resulted in a total of 272,220 contigs reduced to 82,907 after a size and coverage cutoff. Of these 34,560 were annotated and 8,586 submitted to Genbank. Of the 82,907 contigs, 13% where classified as secretory and of the Genbank sequences, 3,882 was classified as secretory proteins and an astounding 564 as lipocalins. A follow up study expanded the read coverage by 315 million additional reads that were combined with the previous studies reads to generate a new assembly from which 25,808 contigs were extracted (Schwarz et al., 2014). The study also included midgut transcriptomes and focused on the first 24 h of feeding. These contigs were used for proteomic analysis of SGE and identified 1,510 proteins from nymphs and adults at 12, 24, and 36 h post-attachment. This is an impressive improvement in proteome coverage compared to number of proteins identified using conventional cDNA libraries. However, the direct digestion of SGE without fractionation may have influenced the total number of proteins detected since crude SGE presents a very high complexity mixture. The transcriptome and proteome data did not correlate with regards to abundance or relative changes, even though biological and technical replicates showed good correlation. Interestingly, the proteomes for the salivary gland and midgut did not differ significantly and ~60% of all proteins did not show any variation in abundance over all time periods sampled. This may reflect that proteomics detect many housekeeping proteins in addition to secreted proteins. Transcriptome levels varied more suggesting transcriptional changes not reflected in the proteome. A sister study classified the same 25,808 contigs according to function and protein family composition as well as for differential expression (Kotsyfakis et al., 2015). This study indicated significant differences in transcript expression profiles between salivary gland and midgut samples. Transcript expression profiles also change over feeding time for different protein families suggesting that “gene switching” occur. It has been suggested that this is a possible way to evade the immune system of the host. In addition, the study indicated higher levels of non-synonymous substitution in secretory proteins suggesting that this indicate higher rates of positive selection.

In a follow up study for *I. ricinus*, transcriptomes from single tick salivary glands were generated (Perner et al., 2018). Salivary glands were sampled from ticks fed on rabbits or artificial membranes at 24, 48, and 72 h. Approximately 435 million Illumina paired reads were generated from 18 libraries and assembled with Illumina reads previously generated in other studies and 40,490 coding contigs were extracted and a final 20,773 contigs with RPKM > 10 were annotated. The study identified 1,907 novel protein sequences of which 406 were identified as secretory. The study indicated that individual ticks show differential expression between ticks and over the course of a blood-meal.

The sialotranscriptomes for three different *Amblyomma* species from Brazil were sequenced using pyrosequencing (Garcia et al., 2014). For *Amblyomma parvum* (Aragão, 1908) 104,817 reads were generated with a final number of contigs of 3,796 of which 493 was annotated as secreted. For *Amblyomma cajennense* (Fabricius, 1787) 67,677 reads were generated with a final number of contigs of 4,604 of which 1,015 was annotated as secreted. For *Amblyomma triste* (Koch, 1844) 442,756 reads were generated with a final number of contigs of 11,240 of which 1,861 was annotated as secreted.

Next-generation sequencing of *A. americanum* sampled unfed, 12, 18, 24, 36, 48, 72, 120, 144, 168, 192, 216, and 264 h after attachment and generated a total of 344,909,378 paired reads (Karim and Ribeiro, 2015). Assembly resulted in a final number of 5,792 contigs that was analyzed. Of these, 2,153 were classified as secretory. Significant differences in transcript levels were observed over the course of feeding, supporting differential expression and the concept of sialome switching was introduced to suggest that the tick switch between different transcriptomes during the course of feeding.

For the zebra tick, *Rhipicephalus pulchellus* (Gerstäcker, 1873) 241,229,128 paired Illumina reads were generated from salivary glands of males and females that were unfed or fed for 1, 3, and 7 h as well as 1, 2, 3, 4, 5, 6, and 7 days (Tan et al., 2015). Assembly resulted in 50,460 coding sequences of which 7,134 was secretory. Proteomics was performed using SGE and *in situ* trypsin digestion followed by LC-MS/MS. This identified 2,231 proteins of which 221 were secretory. Significant differences in transcriptome and proteome expression were observed between males and females.

For *Haemaphysalis flava* (Neumann, 1897), engorged and semi-engorged female salivary glands were used to generate 162,912,848 paired reads, which after assembly yielded a final number of 54,357 contigs (Xu et al., 2015). Of these, 20,145 had homologs in Genbank and 13,513 could be assigned GO terms. Secretory proteins were not reported.

The salivary gland transcriptomes for brown ear ticks, vectors of *T. parva*, the causative agent of Corridor disease, East Coast fever and Zimbabwe theileriosis were described (de Castro et al., 2016, 2017). For *R. appendiculatus*, 430 million paired reads were generated with a final contig number of 21,410 contigs of which 13,996 coded for proteins, of which 2,135 were secretory proteins and 7,414 were annotated as long non-coding RNA. For *R. zambeziensis* (Walker et al., 1981), ~190 million paired reads were generated, assembled and resulted in 140,703 transcripts. After a FPKM > 1 cut-off a final contig number of 23,631 transcripts were obtained of which 15,737 were coding for proteins of which 2,569 were classified as secretory.

For *Amblyomma sculptum* (Berlese, 1888), fed and unfed ticks were sampled and ~567 million single reads were generated (Esteves et al., 2017). Assembly resulted in 9,560 contigs of which 2,314 secretory proteins were found in unfed and 2,592 in the fed transcriptomes. Proteomic analysis identified 124 proteins in saliva of which 58 were secretory. Another study on *A. sculptum*, assembled 195,594,989 Illumina paired reads, 844,529 Ion Torrent reads and 703,210 Roche 454 reads that derived from *Rickettsia amblyommii* infected and uninfected organs that included ovaries, midguts and salivary glands (Moreira et al., 2017). Assembly resulted in 460,445 contigs of which 27,308 were selected for annotation. Of these 2,177 were annotated as secreted salivary gland proteins.

For *Hyalomma excavatum* (Koch, 1844), 138,144,530 Illumina paired reads were generated (Ribeiro et al., 2017). After assembly, 53,228 contigs were obtained and 7,875 coding sequences were annotated with 1,796 classified as secretory. For the camel tick, *Hyalomma dromedarii* (Koch, 1844), male and female ticks at different stages of engorgement were collected from camels in the field (Bensaoud et al., 2018). Illumina sequencing generated 330,285,649 paired-end reads of which 55,819,366 were assembled. After PFAM filtering, 15,342 contigs were annotated of which 1,749 was classified as secretory. The transcriptome study was followed up with proteomic analysis of SGE from ticks collected in the field (Bensaoud et al., 2019a,b). Fractionation of SGE was performed using 1D-SDS-PAGE, after which bands were cut-out, tryptic digested and analyzed using LC-MS/MS. As a target database for the proteomic study, the sialotranscriptome previously generated was used in addition to Acari sequences from Genbank. This identified 1,111 proteins of which 854 were from the Acari database and 257 from the species-specific database. Only 24% of proteins were shared between males and females and 19% of proteins (~211) were classified as secreted.

For *I. holocyclus*, semi-engorged ticks were collected from animals with confirmed paralysis symptoms, before salivary glands were dissected (Rodriguez-Valle et al., 2018). Illumina sequencing generated 65,035,631 paired reads that produced 134,039 contigs after assembly of which ~3,149 was classified as secretory.

For the cattle tick *Rhipicephalus annulatus* (Say, 1821), engorged female ticks that were uninfected or infected with *Babesia bigemina* were used to construct salivary gland transcriptomes (Antunes et al., 2019). Illlumina sequencing generated 40,573,988 paired reads that generated 33,379 contigs in the control and 30,435 contigs in the infected sample of which 16,564 and 15,037 gave significant BLAST hits, respectively. This was reduced to 6,823 and 6,475 unigenes, respectively. Proteomics identified 4,594 proteins in female tick extracts.

For *Amblyomma aureolatum* (Pallas, 1772), transcriptomes were generated from *Rickettsia rickettsii* infected and uninfected tissues (Martins et al., 2019). Approximately 242 million reads were generated of which 110 million were from salivary glands. Assembly generated 11,906 contigs of which 11,903 were expressed in salivary glands.

For the Asian longhorned tick, *Haemaphysalis longicornis* (Neumann, 1901), salivary gland transcriptomes were generated that resulted in 18,313 contigs. The transcriptome was not published, but were used for proteomic analysis with iTRAQ labeling to allow for differential quantification of salivary glands from ~6,000 female ticks that was analyzed as unfed, partially fed, semi-engorged and engorged in three replicates (Ren et al., 2019). Replicate experiments identified 5,059, 5,526, and 5,584 proteins that resulted in 3,667 high-confidence proteins with 2,507 present in all replicates. The majority of proteins identified were housekeeping with significant upregulation of proteins observed during feeding.

A proteomic LC-MS/MS analysis of SGE were performed for the brown ear tick *Rhipicephalus bursa* (Canestrini and Fanzago, 1878) using the Ixodidae database from Genbank (Couto et al., 2020). SGE were prepared from unfed and fed females, which was also either infected, or not with *Babesia ovis*. A total of 1,586 proteins were identified even though only 35 proteins exist in Genbank for this tick species. The majority of proteins were housekeeping, which underscore the utility of sequence databases to identify homologous proteins, but also the impact of the absence of databases to identify species specific proteins.

Giachetto et al. (2020) generated salivary gland transcriptomes for engorged female *R. microplus* that was fed on tick-resistant or tick-susceptible hosts. The transcriptome was assembled from 74,639,552 reads (holstein cattle) and 63,013,658 reads (crossbreed cattle) resulting in 235,451 contigs. Of these 71,757 could be annotated by BLAST analysis but only 1,815 ORFs were extracted of which 20 could be annotated as secretory.

For *R. sanguineus*, female ticks were collected based on weight rather than just days and included unfed, 1.8 mg (day 2), 3.6 mg (day 6), 7.0 mg (day 6), 10.9 mg (day 8), 24 mg (day 8 and 11), 36 mg (days 6, 10, 13), and a sialotranscriptome was constructed (Tirloni et al., 2020). For 20 different libraries that included replicates, 687 million reads were generated from which 71,643 coding sequences were obtained. These were further reduced to 28,921 transcripts with expression values that would represent a minimum of 0.001% of all transcripts based on TPM value (TPM ≥ 10). Of these 8,178 could be annotated with 4,039 identified as secretory proteins. From a similar time series, proteomic analysis of SGE was performed using LC-MS/MS and the transcriptome database generated. This identified 2,125 proteins of that showed a positive correlation between proteome and transcriptome abundance for those transcripts with TPM > 10 (1,745 transcripts). Of 221 with positive correlation, 30% were secretory. A significant number of host proteins (47) were also identified in the SGE. Variability in expression, both in transcript and protein were less marked than previous studies, most probably since the feeding phases of the ticks were better correlated due to weight rather than time. The study again indicated differential expression and the sialome switching phenomenon, both at transcriptome and proteome level.

To date only one salivary gland transcriptome have been described for soft ticks from next-generation sequencing data, namely for *Ornithodoros rostratus* (Aragao, 1911) (Araujo et al., 2019). Salivary glands and midguts were collected from fourth instar nymphs that were unfed or 1 and 4 day fed. Sequencing generated 22,395,831 combined Illumina paired reads that were assembled into 40,058 contigs. Filtering based on ORFs generated a final set of 8,031 contigs that were annotated from which 717 proteins were classified as secretory. In contrast to hard ticks, the secretory proteins in the salivary glands were more abundant than housekeeping proteins and represented 67% of all reads. Another study also sequenced the salivary gland transcriptome of the relapsing fever tick, *Ornithodoros turicata* (Dugès, 1876), but did not describe the transcriptome in a systematic manner (Bourret et al., 2019). The study generated 15,136,406 single-end reads that generated 10,989 contigs after assembly, with 2,138 classified as secretory proteins.

It should be clear from the summary (Table 2) that large variations in data generated, numbers of contigs assembled, final contigs selected for annotation and the final number of contigs submitted to Genbank exists for salivary transcriptomes generated with next-generation sequencing. Given this large variation it may be safe to assume that the transcriptomes for salivary glands may not necessarily be representative of the full complement of proteins expressed in the salivary glands. Additionally, it is not yet clear whether the extremely high number of secretory genes found in the transcriptomes (that increase with each new sampling and sequencing effort) is due to artifacts or a tick-specific mechanism to generate genetic diversity (Mans et al., 2016; Ribeiro and Mans, 2020).

**Table 2:** Statistics for salivary gland transcriptomes assembled using next-generation sequencing technologies.

**Species**	**Total reads**	**Total contigs**	**Contigs after cut-off**	**Annotated**	**HKP**	**SEC**	**Genbank**	**References**
*Amblyomma maculatum*	1,626,969	190,646	72,441	15,814	7,856	3,475	4,849	Karim et al., 2011
*Ixodes ricinus*	68,144,564	272,220	82,907	34,560	19,491	10,777	8,686	Schwarz et al., 2013
*Ixodes ricinus*	~315 million	–	198,504	25,808	12,913	9,048	16,002	Schwarz et al., 2014; Kotsyfakis et al., 2015
*Ixodes ricinus*	~435 million	–	40,490	20,773	–	–	7,692	Perner et al., 2018
*Amblyomma parvum*	104,817	–	–	3,796	2,653	493	2,838	Garcia et al., 2014
*Amblyomma cajennense*	67,677	–	–	4,604	2,805	1,015	5,770	Garcia et al., 2014
*Amblyomma triste*	442,756	–	–	11,240	6,854	1,861	8,098	Garcia et al., 2014
*Dermacentor andersoni*	632,267	21,797	–	21,769	–	–	–	Mudenda et al., 2014
*Amblyomma americanum*	344,909,378	–	–	5,792	3,465	2,153	3,139	Karim and Ribeiro, 2015
*Rhipicephalus pulchellus*	241,229,128	–	–	50,460	11,499	7,134	11,227	Tan et al., 2015
*Haemaphysalis flava*	162,912,848	70,542	55,760	54,357	–	–	–	Xu et al., 2015
*Rhipicephalus appendiculatus*	~430 million	87,688	21,410	21,410	8,237	2,135	20,175	de Castro et al., 2016
*Rhipicephalus zambeziensis*	~190 million	140,703	23,631	23,631	8,139	2,569	21,529	de Castro et al., 2017
*Amblyomma sculptum*	~567 million	–	–	9,560	–	–	4,246	Esteves et al., 2017
*Amblyomma sculptum*	197,142,728	460,445	27,308	27,308	23,248	2,177	–	Moreira et al., 2017
*Hyalomma excavatum*	138,144,530	53,228	–	7,875	5,511	1,796	5,337	Ribeiro et al., 2017
*Hyalomma dromedarii*	55,819,366	142,391	–	15,342	8,063	1,749	142,391	Bensaoud et al., 2018
*Ixodes holocyclus*	65,035,631	134,039	–	134,039	7,975	3,149	95,717	Rodriguez-Valle et al., 2018
*Amblyomma aureolatum*	~242 million	11,903	–	11,903			7,999	Martins et al., 2019
*Rhipicephalus annulatus*	40,573,988							Antunes et al., 2019
*Ornithodoros rostratus*	22,395,831	40,058	8,031	8,031	5,125	717	6,588	Araujo et al., 2019
*Ornithodoros turicata*	15,136,406	–	–	10,989	7,986	2,138	7,560	Bourret et al., 2019
*Rhipicephalus microplus*	137,653,210	235,451		71,757	–	20	–	Giachetto et al., 2020

*Indicated are the total reads generated used in assemblies, the total number of contigs obtained after assembly, number of contigs obtained where a coverage or size cutoff was implemented, the number analyzed for annotation, the number identified as house-keeping (HKP) or secretory (SEC). The number of sequences coding for proteins or contigs deposited in Genbank is also indicated*.

## Next-Generation Sequencing of Whole Body Transcriptomes

Whole body transcriptomes will by default contain salivary gland derived transcripts. In addition, whole body transcriptomes should at least in theory represent the total protein complexity found in a species at the time of sampling and may as such give a better idea on the upper limits of proteins that may be found in salivary gland transcriptomes.

For the ornate cow tick, *Dermacentor reticulatus* (Fabricius, 1794), 7 day unfed larvae were used to construct a transcriptome to investigate stress responses in larvae (Villar et al., 2014). A total of 18,946 transcripts were obtained that were reduced to 3,808 unigenes. Proteomics on larval extracts identified 74, 239 and 104 proteins using various approaches, respectively. The low number of proteins identified may be due to sample complexity or the reduction of proteins to unigenes that likely represented loss of paralogous genes. For *R. sanguineus*, a larval transcriptome was generated for descriptive purposes (De Marco et al., 2017). A total of 5,566,986 short paired-end reads generated 33,396 contigs after assembly and filtering that represented 16,555 unique genes. Dehydration stress in *D. variabilis* was investigated by sequencing unfed male ticks that was dehydrated (Rosendale et al., 2016). From six libraries, 271,494,907 reads were used for assembly that generated 61,800 contigs that were analyzed, of which ~40,000 found BLAST hits. For engorged female *H. longicornis*, ~53 million reads generated 65,916 contigs, of which 23,339 could be annotated (Niu et al., 2019). Whole body transcriptomes for larvae (64,474,326) and nymphs (81,612,022) were also generated (Guo et al., 2019). This resulted in 536,336 transcripts and 440,896 unigenes. Of these 22,347 and 15,112 were annotated by KOG and KEGG databases, respectively. For *I. ricinus*, 15 libraries were constructed for unfed and fed nymphs, unfed males, unfed and fed females (Charrier et al., 2018). From 162,872,698 reads 427,491 contigs were produced that were reduced to a non-redundant dataset of 192,050 contigs. Removal of mammalian and fungal contaminants resulted in 179,316 contigs. Of these 56,809 produced BLAST hits to the Uniref90 or Swissprot databases. Only 12,838 genes were shared with other *I. ricinus* transcriptome studies, while 36,652 were unique to this study and 23,686 was unique to other transcriptomes bringing the potential number of sequences to 73,176 genes. In a follow-up study, the whole body transcriptomes for a number of additional *Ixodes* species were generated for use in phylogenomic analysis using combinations of unfed or fed nymphs, males or females (Charrier et al., 2019). This included *Ixodes acuminatus* (Neumann, 1901) (*n* = 20,250), *Ixodes arboricola* (Schulze and Schlottke, 1930) (*n* = 22,179), *Ixodes canisuga* Johnston, 1849 (*n* = 15,238), *Ixodes frontalis* (Panzer, 1798) (*n* = 18,187), *Ixodes hexagonus* (Leach, 1815) (*n* = 3,215), *Ixodes holocyclus* (*n* = 15,520), *Ixodes uriae* (White, 1852) (49,056), *Ixodes ventalloi* (Gil Collado, 1936) (*n* = 16,563) and *Ixodes vespertilionis* (Koch, 1844) (*n* = 21,090) with the final number of contigs assembled indicated.

Whole body transcriptomes indicate that expected upper limits for salivary gland transcriptomes may range from ~15,000–30,000 genes that can be annotated with our existing annotated databases. One major problem that may exist is overestimation of existing genes due to miss-assembly, such as insertions or deletion not due to exon-intron splicing, or extension of 5′ or 3′ ends that cause such genes to be identified as unique even though the rest of the gene is 100% identical to the canonical gene. While existing algorithms may identify and remove chimeric transcripts (miss-assembly due to linkage of two canonical genes or fragments), identifying miss-assembled transcripts due to small inserts, deletions or extensions is more difficult with existing algorithms and require extensive manual curation.

## Proteomics of Tick Saliva

Apart from the studies mentioned above that specifically focused on salivary gland transcriptome descriptions with added proteomic analysis, some studies focused more specifically on the analysis of tick saliva using proteomics (Table 3). The salivary transcriptome of *D. andersoni* were sequenced using Roche 454 technology for the purpose of proteomic analysis (Mudenda et al., 2014). A total of 632,267 reads were generated at three time points (Day 0, Day 2, and Day 5). Assembly yielded 21,769 unique sequences coding for proteins. Each time point presented unique transcripts as well as up- or down-regulation of genes during the course of feeding. The study also collected saliva for day 2 and day 5 of feeding and fractionated this with 2D chromatography (in-line cation exchange linked with reversed phase) yielding 30 fractions analyzed by MS/MS. An impressive number of 677 proteins were detected.

**Table 3:** Summary of proteomic analysis for tick salivary gland proteins using various technologies.

**Species**	**Extract type**	**Technology**	**Tick Proteins**	**Percentage of Genbank**	**Species specific sequences in Genbank/Study**	**HKP**	**SEC**	**References**
*Ixodes scapularis*	SGS SGE	Edman Edman	12 11	13.7 12.6	87	0 4	12 7	Valenzuela et al., 2002a
*Ornithodoros erraticus*	SGE	MALDI-MS	6	–	0	5	2	Oleaga et al., 2007
*Ornithodoros moubata*	SGE	MALDI-MS	2	4.1	48	0	2	Oleaga et al., 2007
*Ornithodoros parkeri*	SGE	Edman 1D-LC-MS 2D-LC-MS Total	12 29 11 37	7.6 18.3 6.9 23.4	158	0 0 2 2	12 29 9 35	Francischetti et al., 2008a
*Ornithodoros coriaceus*	SGE	Edman 2D-LC-MS 1D-LC-MS Total	3 7 36 39	2.8 6.6 34.3 37.0	105	0 0 0 0	3 7 36 39	Francischetti et al., 2008b
*Argas monolakensis*	SGE	Edman 2D-LC-MS LC-MS Total	14 14 28 35	7.2 7.2 14.5 18.1	193	0 0 0 0	14 14 28 35	Mans et al., 2008
*Rhipicephalus sanguineus*	SGS	1D-LC-MS	21	9.7	217	12	9	Oliveira et al., 2013
*Amblyomma variegatum*	SGE	1D-LC-MS	170	28.0	605	151	19	Ribeiro et al., 2011
*Hyalomma rufipes*	SGE	1D-LC-MS	72	73.4	98	75	23	Francischetti et al., 2011
*Ornithodoros moubata*	SGS	LC-MS	193	257.3	75	72	3	Díaz-Martín et al., 2013
*Dermacentor andersoni*	SGS	LC-MS	677	3.1	21,797	–	–	Mudenda et al., 2014
*Rhipicephalus microplus*	SGS	1D-LC-MS	187	–	22,009	41	145	Tirloni et al., 2014
*Haemaphysalis longicornis*	SGS	1D-LC-MS	135	–	–	106	29	Tirloni et al., 2015
*Ixodes scapularis*	SGS	1D-LC-MS	582	–	62,246	–	–	Kim et al., 2016
*Amblyomma americanum*	SGS	1D-LC-MS	1,182	–	110,587	–	–	Kim et al., 2020

*Protein mixtures analyzed were obtained via induction of salivary gland secretion (SGS) or salivary gland extracts (SGE). Mixtures were analyzed by one-dimensional SDS-PAGE followed by blotting and Edman sequencing (Edman), matrix-assisted laser desorption ionization (MALDI-MS), one-dimensional SDS-PAGE from which bands were cut out for tryptic digest followed by MS/MS analysis (1D-LC-MS), or two-dimensional SDS-PAGE from which bands were cut out for tryptic digest followed by MS/MS analysis (2D-LC-MS), or salivary gland proteins fractionated using liquid chromatography followed by MS/MS (LC-MS)*.

Tirloni et al. (2014) performed proteomic analysis on saliva collected from partially and fully fed *R. microplus*. For this, saliva was fractionated on 1D-SDS-PAGE and 42 bands for partially engorged and 15 bands for fully engorged was excised, in-gel trypsin digested and analyzed on LC-MS/MS. Saliva was also directly digested *in situ* before LC-MS/MS analysis. Spectra were analyzed against an in-house database of 22,009 protein sequences. The study identified 187 tick and 68 host proteins. Another study that also compared saliva collected from partially and fully fed *R. microplus* by iTRAQ labeling followed by LC-MS/MS analysis, identified 322 unique proteins of which 41 was considered high-confidence and was found in both partially and fully fed samples (Feng et al., 2019).

Using a similar approach to Tirloni et al. (2014), performing *in situ* trypsin digestion of saliva from nymphs and adults from *H. longicornis*, followed by LC-MS/MS analysis, 135 tick and 100 rabbit proteins were identified (Tirloni et al., 2015). The authors used the in-house salivary gland transcriptome database of 22,009 sequences from *R. microplus* and the Ixodidae sequences from Genbank. In a study using a similar methodology that collected saliva from *I. scapularis* females at 24, 48, 72, 96, and 120 h as well as engorged and detached, a total of 769 tick and 130 rabbit proteins were identified (Kim et al., 2016). The NCBI non-redundant database (62,246 Ixodidae entries) were used for identification, rather than a species-specific database, since this database presumably presents the most up to date collection of *I. scapularis* sequences derived from both the genome and transcriptomes. The authors indicate that protein profiles change over the course of feeding and suggest that this switching mechanism is used as immune evasion strategy. More recently, the saliva profile of *A. americanum* was determined using the *in situ* digestion, LC-MS/MS approach (Kim et al., 2020). Saliva was collected from ticks attached for 24, 48, 72, 96, 120, 144, 168, and 192 h, as well as engorged and detached. The database used was from sequences generated previously (Radulović et al., 2014). This transcriptome was reassembled from the datasets and generated 110,587 contigs used for analysis. This study identified 1,182 tick and 335 rabbit proteins.

From these studies, saliva complexity at any given point seems low and corresponds with roughly 200 proteins. Complexity increase with number of sampling points, since protein expression patterns change over time, but it would seem, also with the size of the species-specific database. With regard to sampling points, the current practice is to sample at 24 h intervals and protein expression patterns may change between sampling events, suggesting that change can occur within hours. At yet, it is not clear how fast this change can occur. Even so, not all proteins identified are secretory, but many are housekeeping and some host-derived (Tables 1, 3). The numbers identified still do not correlate with the almost 10-fold larger number of transcripts identified in the transcriptomes (Mans et al., 2017). This would suggest that transcriptomes generate an over-estimation of complexity or that proteomic analysis are not yet technologically advanced to identify all proteins present in complex salivary mixtures.

**Figure 2:**
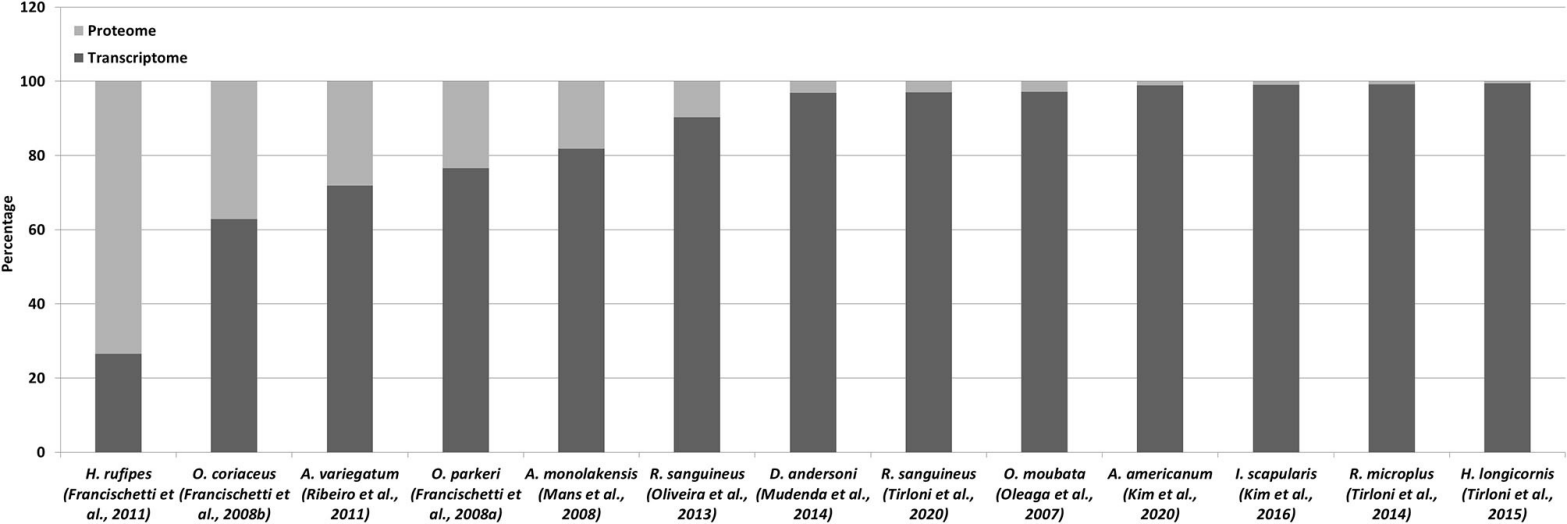
Proteomics of tick transcriptomes. Indicated is the percentage of proteins identified using proteomics relative to the transcriptome database used for identification.

The above sections detailed both transcriptome and proteomic studies since these are generally closely linked. It has been indicated that species-specific transcriptomes are essential for detection of lineage specific proteins and to increase proteome coverage. Even so, the use of proteomes to validate transcriptomes is just as valid. In a perfect world, each contig produced by a transcriptome sequencing project should be confirmed and validated by proteomic analysis, since proteins represents functional entities (Mans, 2019). However, the coverage of transcriptomes by proteomes has been dismal to date (Figure 2). In the case of transcriptomes derived from conventional cDNA libraries the transcriptome coverage detected by proteomics was decent, ranging from 10 to 73% (Table 1; Figure 2). This could be explained by the representation of highly abundant transcripts in relatively small transcriptomes, correlating with highly expressed proteins in the proteome. As transcriptome sizes increased, the number of proteins detected in the proteome also increased, but not at the same ratio. As such, while proteins detected by proteomics may range in the thousands, the percentage of transcriptome coverage is lower compared to conventional cDNA derived transcriptomes (Table 3). The potential reasons for this are numerous. Proteomic technology may not yet be sensitive enough to obtain the depths attained by next-generation sequencing of transcriptomes. This may be linked to both hardware limitations of mass spectrometry equipment, but also to sample fractionation strategies. In regard to the latter, the more complex the sample, the lower the expected coverage that may be obtained. Fractionation of samples that decrease sample complexity but retain yields for low abundance proteins should yield higher detection numbers for proteins. The number of time points sampled for proteomics may impact on the number of proteins identified, with more sampling points yielding more proteins. Transcriptomes may present artifacts, i.e., transcripts that do not represent proteins present in a given sample. This may be due to misassemblies or transcription of pseudogenes that are not translated into protein (Mans et al., 2017).

## Proteomics of Tick Cement

An integral part of salivary complexity is the cement cone produced by some *Ixodes* and metastriate ticks (Chinery, 1973; Suppan et al., 2018). These are proteins secreted in soluble form in the saliva that then harden to a matrix that can anchor the tick to the host during feeding. Analysis of the cement cone was historically difficult due to its insolubility. However, the use of chaotropic agents such as urea and SDS to solubilize the cement cone, followed by proteomic analysis have allowed a deeper insight into cement composition. For *A. americanum*, cement were dissolved in 8M urea followed by SDS-PAGE, before bands were cutout for proteomic analysis. This identified 7 proteins that included a glycine-rich and metalloprotease as well as proteins considered housekeeping using the *A. americanum* salivary transcriptome and tick database (Karim and Ribeiro, 2015; Bullard et al., 2016). A follow up study solubilized the cement cone in 8M urea, after which alkylation and tryptic digestion were followed with LC-MS/MS identification. This identified 160 proteins using an in-house *A. americanum* database (Porter et al., 2015; Hollmann et al., 2018). The proteins identified included as major proteins glycine-rich proteins and protease inhibitors as well as a large proportion of house-keeping proteins. A more recent study identified 654 proteins in urea+SDS extracted vs. 388 proteins in SDS vs. 266 proteins in urea alone from cement cones of *R. microplus* (Villar et al., 2020). The protein profile from cement was also termed the “cementome.” The study also identified 2,264 proteins from salivary gland extract. Of the proteins identified in the cement only 81 seem to be tick-derived while the rest were host-derived. The tick proteins were composed of glycine-rich proteins, protease inhibitors and various enzymes proposed to play a role in cement formation, solidification and maintenance. Complexity of the cement cone therefore seem to increase as extraction methodologies improve. A question that obviously remain is how many proteins may be entrapped during cement formation that do not play a primary role in the cement plug.

## The Completeness of Sialotranscriptomes

Next-generation sequencing technologies raised the problem of what would be considered to be true in transcriptomics. In all publications published to date several recurring issues may be observed. Assemblies lead to large numbers of contigs and cut-off values needs to be implemented to reduce the number of contigs analyzed. This is generally based on coverage, either RPKM or TPM values or a subjective selection of the cutoff value. While this result in more trustworthy final assemblies, it raises the question of the origin of those contigs that do not make the selection criteria. Are these assembly artifacts, background transcription artifacts, environmental contamination or contamination from other tick tissues?

**Figure 3:**
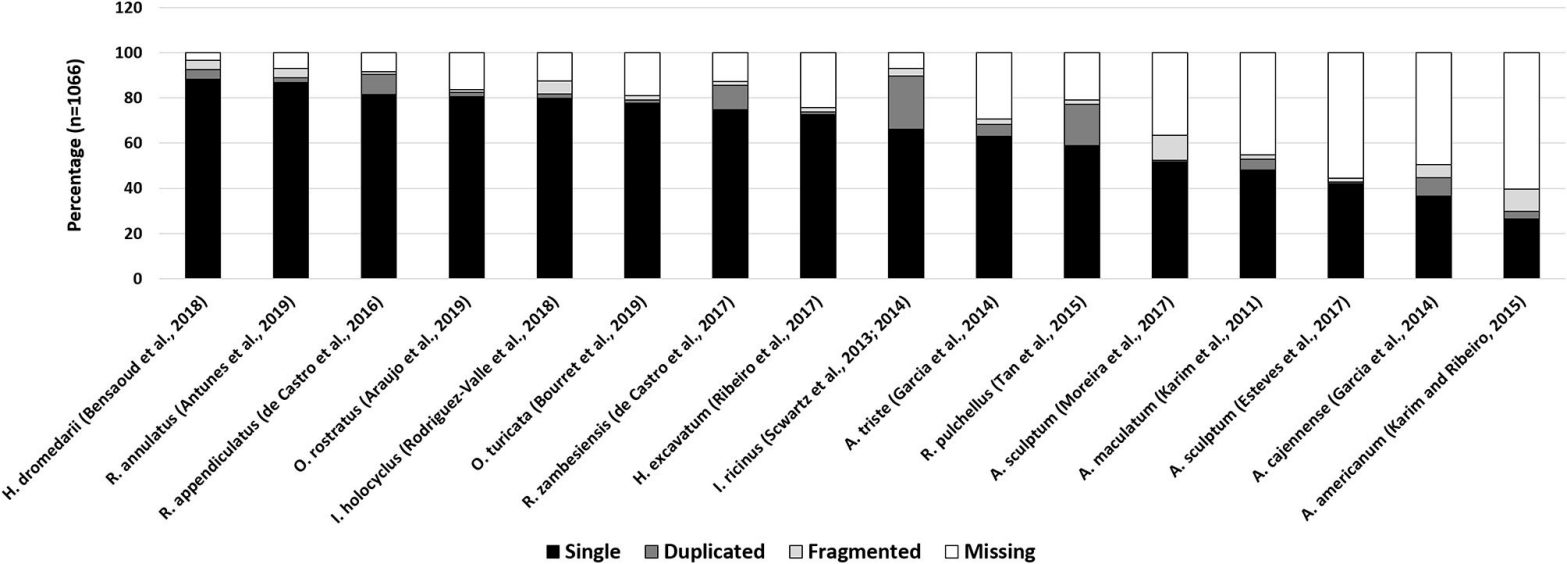
BUSCO analysis of tick salivary gland transcriptomes. Indicated are various published tick transcriptomes and their BUSCO analysis. Complete genes were detected as single copies (single) or multiple copies (duplicated). Genes that are fragmented or missing are also reported.

To determine how representative a transcriptome is, the presence of expected household genes can be determined. The “completeness” of this set may then be used as a proxy for the quality of a transcriptome. The program “Benchmarking Universal Single-Copy Orthologs or BUSCO” uses the concept of universal genes that are present as singletons in both genomes and transcriptomes (Waterhouse et al., 2018). BUSCO analysis of all published salivary gland transcriptomes generated with next-generation sequence technologies, indicate that the “completeness” of these transcriptomes ranges from 40 to 97% (Figure 3). This would imply that salivary transcriptomes have not been sequenced to depths necessary to recover all genes. However, as indicated above, the BUSCO scores may also have been influenced by the cut-off values used in post-assembly analysis. While universal genes may certainly be indicative of how well housekeeping genes may be represented in transcriptomes, their function as proxy for the completeness of secreted protein families should be considered with caution. Small, abundant, multi-copy, polymorphic families such as the BPTI, BTSP, and lipocalins, may present their own problems during assembly since these genes are likely to be lost during assembly due to non-uniform sequence coverage and assembly artifacts. As such, while salivary gland transcriptomes have been expanded by NGS technologies the results suggest that we may still be missing a third or more of the transcriptome.

## Complexity Measured by Genome Sequencing

While transcriptome sequencing can certainly give insight into gene diversity, the above discussion indicate that it may not be possible to derive at a final number of genes using transcriptomics. The gold standard for definitively estimating gene numbers remains whole genome sequencing. For ticks, draft genomes of *I. scapularis, I. ricinus, H. longicornis*, and *R. microplus* has been published (Cramaro et al., 2015, 2017; Gulia-Nuss et al., 2016; Barrero et al., 2017; Miller et al., 2018; Guerrero et al., 2019).

The first genome for *I. scapularis* was sequenced using Sanger sequencing (Gulia-Nuss et al., 2016). The estimated genome size was 2.1 Gb with 20,486 predicted genes. BUSCO analysis indicated 69% completeness based on 2,675 reference genes (Mans et al., 2017). The ISE6 cell line from *I. scapularis* was also sequenced (Miller et al., 2018). This estimated the genome size at 2.8 Gb but if duplications are taken into account the size reduces to 2.2 Gb. BUSCO analysis indicated 95% completeness for 1,066 reference genes. The total number of genes associated with this genome is 37,259 with 24,501 protein coding. However, it has been estimated that as much as 50% of salivary gland proteins may be missing or incomplete in the genome (Ribeiro and Mans, 2020).

The genome for *I. ricinus* has been sequenced with both Illumina and Pacific Biosciences (PacBio) long read technologies (Cramaro et al., 2015, 2017). The estimated size of the genome is 2.65 Gb from which 516 Mb were sequenced which represent 19.4% of the genome but 67% of the non-repetitive genome. A total of 25,263 proteins were identified based on BLAST similarity searches. However, BUSCO only indicated 55.5% completeness.

The genome for *H. longicornis* was sequenced using PacBio long read technology (Guerrero et al., 2019). Genome sequencing was facilitated by the parthenogenicity of this species (ability to asexually reproduce with mating) that reduce genome heterogeneity. The total length of the genome was 7.3 Gbp in 34,208 contigs and was 96% complete as assessed with BUSCO. To date a gene count is not available.

The genome of *R. microplus* was sequenced using an Illumina/PacBio hybrid assembly approach (Barrero et al., 2017). Given the highly repetitive nature of the genome (70%) and the large estimated size (7.1 Gbp), Cot filtration was used to enrich for single, low copy, and moderately repetitive genomic DNA. This yielded an assembly of 2 Gbp with 38,827 genes of which 24,758 is protein coding. BUSCO analysis indicated a completeness of 53.1%. Conversely, a 63,416 non-redundant transcriptome dataset showed 85% completeness, suggesting that the genome assembly still miss 47% proteins encoded in the genome.

While all available tick genomes yield protein coding genes ranging from ~20,000 to 30,000 it is clear that their incomplete nature also do not allow accurate estimations of complexity of the whole genome, nor what may be expected at the tick-host interface.

## Complexity Measurements Only as Good as the Existing Databases

It has been indicated that database repositories for sequences has an immense impact on the coverage and complexity detected during proteomic analysis, but also impact on the ability to annotate transcriptomes. In this regard, tick sequences are scattered across many different databases, none which present a complete non-redundant set of sequences. For VectorBase (Giraldo-Calderón et al., 2015), the only datasets available is for *I. scapularis, I. ricinus*, and *R. microplus* which is effectively those for which genome datasets exist. For Genbank, the nucleotide core database contain the majority of historical sequences deposited in Genbank, but only links to transcriptome shotgun assembly (TSA) sequences (that include EST and next-generation sequencing assemblies contained in the small read archive database -SRA). For many tick transcriptomes, data is only available in the small read archive database (SRA) as contigs (that may or may not contain full length ORFs) and no protein or nucleotide coding sequences exist. In some instances, no contigs were deposited and only raw sequence read data is available. This generally necessitates either extraction of ORFs or reassembly and annotation to make this data useful for comparative or proteomic analysis, although given the diversity of methods available for transcriptome assembly and analysis, the reproducibility of constructing a published transcriptome is debatable. In many cases as indicated, researchers use in-house sequence databases not deposited anywhere and therefore not available. There is therefore a need for a centralized database that would address all of these shortcomings and would include comprehensive inclusion of all tick transcriptomes sequenced to data as well as accurate annotation. To compound this, the unique lineage specific expansions observed in ticks make extraction of functionally relevant information difficult since proteins may only be annotated as “potential secretory protein” or “unknown function” (Ribeiro and Mans, 2020). In many cases, such proteins cannot even be assigned to existing protein families. In an attempt to partially address this, a database of secretory proteins has recently been constructed that can be searched by reverse position specific BLAST to assign potential secretory proteins to known secretory families (Ribeiro and Mans, 2020). However, this database only attempted classification of those proteins present in the NCBI non-redundant sequence database and excluded TSA and SRA databases. The searchable database will however, allow for better annotation of transcriptomes using a consistent nomenclature.

With regard to proteomic analysis, the data described in publications in tables or supplementary materials is generally useful to document the snapshot provided by a specific study. However, the main usefulness of proteomic data lies in the re-analysis potential of the raw data once sequence databases has been expanded since this may result in higher numbers of proteins identified from the proteomic data. To this end, raw proteomic data can be submitted to the PRoteomics IDEntification database (PRIDE) (Perez-Riverol et al., 2019). To this end, proteomic data for at least 6 tick species is available in PRIDE and include *A. americanum* (Crispell et al., 2019; Kim et al., 2020), *A. sculptum* (Esteves et al., 2017), *I. scapularis* (Villar et al., 2015; Kim et al., 2016), *I. ricinus* (Cramaro et al., 2015), *H. longicornis* (Ren et al., 2019), and *R. sanguineus* (Tirloni et al., 2020).

## Other Measures of Complexity: Alternative Splicing, Recombination, Non-Coding RNA and Posttranslational Modification

The current study has thus far largely focused on detection and quantification of genes and the proteins encoded by them, as measures of salivary gland complexity. The impact of exon shuffling and gene duplication has not been considered yet, since these are mechanisms to generate diversity during long-term evolution which is detected retrospectively (Mans et al., 2017). However, alternative splicing may contribute significantly to increase complexity since this may be tissue specific and give rise to protein isoforms (Nilsen and Graveley, 2010). The impact of alternative splicing on complexity has not yet been analyzed in depth in ticks or salivary gland products. For example, the search terms “alternative splicing” and “ticks” retrieve 11 hits in PubMed. Of these, 9 deals with potential alternative splicing in single genes, the majority which is housekeeping (Baxter and Barker, 1998; Guo et al., 1998; Saravanan et al., 2003; Xu et al., 2003; Tabish et al., 2006; Buresova et al., 2009; Olafson et al., 2011; Temeyer et al., 2012; Urbanová et al., 2018). None of the genome, proteome or transcriptome studies described in the current study considered alternative splicing or isoforms extensively, except for the study of Oleaga et al. (2007) that detected numerous isoforms on a two-dimensional gel for the histamine and serotonin-binding protein, TSGP1. It is unclear whether these isoforms were due to alternative splicing or post-translational modification. The absence of extensive detection of alternative splicing in salivary gland proteins may be partially due to the consideration that most secretory tick families are single domain proteins such as the BPTI, BTSP, cystatin or lipocalin families, or composed of multiple domains of these core folds such as for example the various BPTI proteins with double, triple, quadruple and quintuple domains, or composed of multi-domain proteins with well-defined domain structures such as 5'-nucleotidase and the metalloproteases (Francischetti et al., 2009). On this, large scale proteomic studies support the observation that a single main isoform exist for most proteins and that other isoforms may not be functional, or under selective pressure or even expressed (Tress et al., 2017), while splicing of whole domains is also unlikely (Light and Elofsson, 2013). The potential for alternative splicing, exon shuffling and intragenic recombination to generate “new genes on the fly” has been considered to explain the sheer diversity of secretory proteins observed in the salivary glands (Ribeiro and Mans, 2020). Real data is, however, needed to confirm this.

A further level of complexity that may be emerging is that of non-coding RNA. Abundant long non-coding RNA was found in tick transcriptomes (de Castro et al., 2016), while microRNA may perform important housekeeping functions (Barrero et al., 2011; Zhou et al., 2013; Luo et al., 2015, 2019; Shao et al., 2015; Malik et al., 2019; Liu et al., 2020). However, of more interest for the current study is the potential that some of these non-coding RNA may play a role in the regulation of host defense mechanisms. As such, exosomes that carry microRNAs has been described in the saliva of ticks (Hackenberg et al., 2017). It has been proposed that these exomes may deliver microRNAs to immune cells to down-regulate immune modulatory pathways (Hackenberg and Kotsyfakis, 2018; Bensaoud et al., 2019b; Chávez et al., 2019). This will add another layer of complexity to salivary glands of which the magnitude is difficult to estimate. As yet, down-regulation of such immune pathways needs to be proven and it needs to be shown that concentrations of these microRNAs can break the chemical equilibrium barrier to be effective (Mans, 2019).

Post-translational modifications play a large role in regulation of functional networks and in protein function. With regard to protein function involved in tick-host interaction a number of post-translational modifications have been shown to be important for functionality, especially tyrosine sulfation (Shabareesh et al., 2017; Thompson et al., 2017; Vechtova et al., 2018; Franck et al., 2020). Many of the transcriptome studies listed also report many potential modification sites or motifs. However, large scale confirmation of post-translational modifications by proteomic analysis has not yet been performed for ticks (Vechtova et al., 2018). As such, the impact of post-translational modifications on complexity of salivary gland proteins is as yet unknown, but could be quite significant.

## Future Perspectives on Salivary Gland Complexity

We are undoubtedly much further along the road to describing and understanding tick salivary gland protein complexity than ever before. It is likely that the next 10–20 years will see the completion of many tick genomes, the validation of many tick transcriptomes by proteomics and final estimates on the complexity of salivary gland protein repertoires for many tick species. This will be facilitated by new technologies such as third generation nanopore sequencing that will hopefully make transcriptome and genome sequencing affordable for the high number of species required to map all orthologs and paralogs. Other challenges are defined by our ignorance of the mechanisms regulating sialome switching, and the mode and possible physiological role of host protein secretion by tick salivary glands. The next challenge will be to analyze all this information to generate a comprehensive, holistic and ultimately understandable synthesis of tick evolution and tick-host interaction. Even so, we may find out that many genes identified in the genomes or transcriptomes may never be validated by proteomics. This may be due to the presence of pseudogenes or expression so transient to remain undetectable by proteomics, or even be functionally irrelevant (Mans et al., 2017; Mans, 2019). In the end, validation by empirical testing will remain crucial.

As indicated, the salivary gland is a complex organ composed of multiple acini with each comprising multiple cell types. While temporal differential expression over the course of feeding has been well-characterized, differential expression also occur within different cell types and acini. To define this expression well is important, since some cells are prone to infection by pathogens, such as *T. parva* that infect the e-cells of type III acini (Fawcett et al., 1982). In this case the impact of parasite infection on vector expression (Nene et al., 2004), is confounded by non-infected/non-affected members of the same cell type and other cell types since the parasite do not infect all cells of a particular type at the same time (McKeever, 2009). Other cell types are involved in specific biological functions such as water secretion by the e-cells of type III acini (Fawcett et al., 1981). An accurate picture of global differential expression is therefore confounded by the differential expression that occur on smaller scales. The potential to address this issue exist via single cell sequencing approaches (Chattopadhyay et al., 2018). These approaches are hampered by the pyramidal structure of acini and the fragility of salivary gland cell types, but future innovations on non-disruptive separation of acinar cell types may enable single cell sequencing. Alternatives may also be found in laser capture microdissection of cell regions followed by single cell sequencing (Chattopadhyay et al., 2018). It is foreseen that these approaches will certainly enable a deeper dissection of salivary function, localization and parasite-vector interaction.

The majority of studies described for salivary gland transcriptomics are for adult ticks, while whole body transcriptomes are generally generated for larvae or nymphs. Single cell and related technologies that allow sequencing of minute quantities of genetic material may open up the possibility of sequencing larval or nymphal glands made accessible via laser microdissection. This will certainly advance tick salivary gland transcriptomics, since differential expression between life stages is well-recognized (Schwarz et al., 2013; Andreotti et al., 2018).

## Author Contributions

The author confirms being the sole contributor of this work and has approved it for publication.

## Conflict of Interest

The author declares that the research was conducted in the absence of any commercial or financial relationships that could be construed as a potential conflict of interest.
